# Case Report: Difficulties faced by a home oxygen therapy patient who died after the Fukushima Daiichi nuclear power plant accident

**DOI:** 10.3389/fpubh.2024.1394376

**Published:** 2024-07-16

**Authors:** Momoka Yamamura, Toyoaki Sawano, Akihiko Ozaki, Tianchen Zhao, Chika Yamamoto, Stephanie Montesino, Moe Kawashima, Yuna Uchi, Hiroki Yoshimura, Kemmei Kitazawa, Hidenori Marui, Masaharu Tsubokura

**Affiliations:** ^1^Department of Radiation Health Management, Fukushima Medical University, Fukushima, Japan; ^2^Department of Surgery, Jyoban Hospital of Tokiwa Foundation, Fukushima, Japan; ^3^Research Center for Community Health, Minamisoma Municipal General Hospital, Fukushima, Japan; ^4^Breast and Thyroid Center, Jyoban Hospital of Tokiwa Foundation, Fukushima, Japan; ^5^Icahn School of Medicine at Mount Sinai, New York, NY, United States; ^6^Shirakawa Kosei Hospital, Shirakawa, Japan; ^7^School of Medicine, Hiroshima University, Hiroshima, Japan

**Keywords:** home oxygen therapy, disaster-related death, disaster medicine, emergency preparedness, Fukushima nuclear accident

## Abstract

Following the Great East Japan Earthquake (GEJE) and the Fukushima Daiichi nuclear power plant accident in 2011, mandatory evacuation orders were issued to residents living near the nuclear power plant in Fukushima prefecture, including some patients receiving home oxygen therapy. Although the vulnerability of patients with home oxygen therapy (one of the population groups most vulnerable to disasters) has been noted, there is little information on the health effects of evacuation in the event of a radiation disaster. A 90-year-old man diagnosed with chronic obstructive pulmonary disease since the age of 70, and lived in a town located approximately 20 km south of the nuclear power plant, died 8 months after the disaster due to worsening health conditions. This case reveals the potential for both physical and psychological burdens experienced by vulnerable groups like patients undergoing home oxygen therapy during evacuations in times of disaster. Although it is only a case report and the information is limited, severe respiratory distress requiring home oxygen therapy may present a significant risk factor for disaster-related deaths, especially in cases where evacuations are prolonged, such as in nuclear disasters. Due to the challenge of obtaining prompt public support immediately after a disaster, home oxygen therapy patients may need to prioritize self-help and mutual assistance in their disaster preparedness efforts.

## 1 Introduction

In recent years, there has been an increase in the number of disasters worldwide (1). In the event of a disaster, individuals with pronounced health vulnerabilities—who face greater challenges in being evacuated independently compared to the general population and require assistance doing so—are referred to as “disaster-vulnerable individuals” (2). Thus, preparedness for disasters prioritizing them is essential. Disaster-vulnerable individuals include infants and children (3), individuals receiving home medical care, older adults with cognitive decline or physical frailty, those with mental and physical disabilities, and foreigners with limited local language proficiency and low social status (4). In particular, the older adults, individuals with disabilities, and those receiving home medical care are at a significantly greater risk of compromised health, even with minor environmental changes (5–10). While the deaths of those who perish because of disasters are called disaster deaths, the deaths of those who pass away indirectly due to the aftermath are called disaster-related deaths. Vulnerable population groups are the most susceptible to disaster-related deaths. Consequently, advanced disaster planning is necessary to mitigate the impact of disasters disaster-stricken population (11, 12). As disasters become more frequent and larger in scale, there is an urgent need to prepare vulnerable population for crises to minimize disaster-related deaths.

In this context, patients receiving home oxygen therapy, who are primarily in the terminal stages of chronic obstructive pulmonary disease (COPD), are worth focusing on given their vulnerability. It is a respiratory illness characterized by prolonged exposure to harmful substances like tobacco smoke and silicon dioxide. This may produce inflammation in the lungs and trachea, gradually causing symptoms such as shortness of breath, coughing, and phlegm. This can ultimately lead to respiratory failure and death. As the illness progresses and reaches an advanced stage, some patients require regular oxygen support on a daily basis, leading to indications for home oxygen therapy. Home oxygen therapy has been shown to prolong prognosis in patients requiring oxygen; and its discontinuation may lead to an increase in hospitalization or mortality, potentially worsening the prognosis (8, 13). The vulnerability of patients with COPD to disasters is illustrated in prior studies (14, 15). It has been found that vulnerability during disasters arises from factors like insufficient oxygen production at home due to power outages as well as shortages of portable oxygen for evacuation, all of which can significantly affect patients' health (8). From the experience of past disasters, it has been reported that the health conditions of patients with respiratory diseases deteriorate, causing hospitalizations to increase during the acute phase of disasters (16, 17). However, there is limited information specifically regarding how the health of those with home oxygen therapy is impacted during disasters.

In March 2011, following the Great East Japan Earthquake (GEJE) and the subsequent Fukushima Daiichi Nuclear Power Plant disaster, the residents of the Hamadori region in Fukushima Prefecture were ordered to evacuate. Many vulnerable individuals were forced to evacuate. Consequently, the number of disaster-related deaths certified in Fukushima Prefecture alone exceeded 2,300 people (18). This tragedy highlights the significance of providing support for vulnerable populations in the aftermath of disasters (19–26). This includes individuals receiving medical treatment at home (such as those undergoing home oxygen therapy, who were not exempt from evacuation). They were compelled to leave while seeking medical resources and faced a constant threat to their lives throughout the entire evacuation process. The vulnerability of home oxygen therapy patients, one of the groups most susceptible to health impacts during disasters, has been previously pointed out (27). Reports have long underscored the impact of insufficient home oxygen production and the shortage of portable oxygen for evacuation during power outages caused by disasters on patient health (28). In the areas affected by the GEJE, surveys have been conducted to investigate the evacuation plans of patients with home oxygen therapy (29). Nevertheless, to date, limited information has been available on how evacuation affects the health of home oxygen therapy patients when a disaster strikes, particularly regarding the health effects of the physical and psychological burdens faced by such patients during evacuations resulting from radiation calamities.

Here, we report the case of a 90-year-old man who was receiving home oxygen therapy and whose prognosis was likely affected by the prolonged evacuation resulting from the Fukushima Daiichi Nuclear Power Plant nuclear accident. Documenting and interpreting this case are valuable for assessing the effects of evacuations on individuals with physical disabilities at home (such as home oxygen therapy patients) and may prove useful in minimizing the impact of evacuations during future disasters.

## 2 Case presentation

The case of a 90-year-old man with a history of COPD, who had been diagnosed around the age of 70, resided in the town of Naraha, located approximately 20 km from the Fukushima Daiichi Nuclear Power Plant was studied. Despite his need for continuous 24-h oxygen supplementation, he maintained a degree of independence in daily activities such as short-distance walking, standing, sitting, and managing tasks such as meal preparation, toileting, maintaining oral health care, dressing, and bathing. His predominantly indoor lifestyle was occasionally punctuated by outdoor ventures, utilizing an electric car to explore nearby fields and farms, providing him with joy as he observed the growth of rice and vegetables.

The seismic events of GEJE struck Japan on March 11th, 2011, inflicted damage to the patient's house, prompting his family to evacuate and reach a community center in Naraha. Following the subsequent Fukushima Daiichi Nuclear Power Plant nuclear accident on March 12th, all residents were ordered to evacuate, leading the patient and his family to take refuge in the gymnasium of an elementary school in Iwaki, approximately 40 km south of his residence. Despite carrying a portable oxygen tank and using their car, the journey that would typically take 30 min extended to 4 h due to congested roads and challenging terrains. This marked the beginning of a grueling evacuation for the patient, unaccustomed to such prolonged transfers.

The designated evacuation site, the elementary school gymnasium, was crowded and frigid, prompting the patient and his family to seek refuge with a relative in Iwaki. The room provided, seldom used and cold, made for an uncomfortable night for the patient. Typically, a portable oxygen cylinder can be set from 0.5L/min to 10L/min, and the patient had required 1.0L/min at the time. Amidst the chaos, they carefully managed the oxygen supply to prevent it from running out, continually securing oxygen from suppliers during the evacuation process. Furthermore, they reached out to a supplier, who delivered oxygen to their evacuation site, enabling them to secure the necessary oxygen and keep him alive. As air radiation levels rose in Iwaki on March 15th, the family evacuated to Tokyo on March 15^th^ with two portable oxygen tanks, enduring a 13-h car journey. They reached a relative's house in Tokyo on March 16th, and stayed there until March 25th. The place where he had put up was that of one of his relatives', who also received home oxygen therapy, so he was able to borrow their oxygen device.

**Figure 1:**
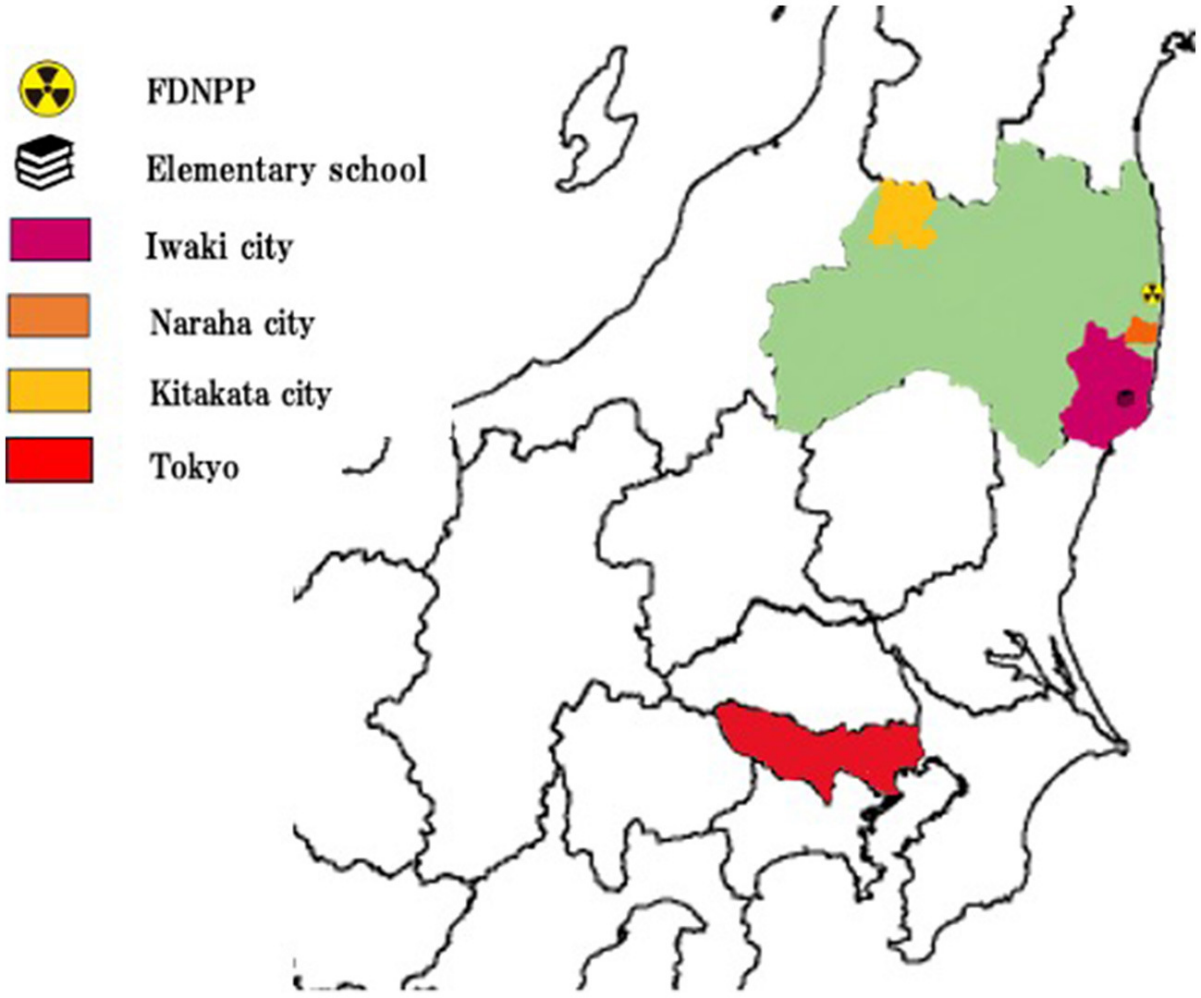
Map of the patient displacement: routes of evacuation across Fukushima prefecture.

On March 25th, the family rented an apartment in Tokyo, but the patient's health continued to decline. During this period, the patient developed a fever close to 40 degrees Celsius, leading to a hospital visit. He was admitted to a hospital in Tokyo on April 19th with respiratory distress and fever. According to the medical records, the patient was diagnosed as experiencing physical debilitation and worsening respiratory distress due to evacuee living conditions, resulting in a state of immobility. Upon admission, the patient's respiratory condition was significantly compromised, prompting the physician to suggest intubation. However, the family decided against intubation. Ultimately, the patient's respiratory condition improved without intubation. Although he was able to walk on his own at the initiation of the evacuation, his condition worsened, requiring all assistance for daily activities and eventually led to the use of diapers at the admission. Due to his decreased muscle strength, it also became difficult for him to undergo rehabilitation. Upon his expressing a strong desire to return to Fukushima, he was transferred to a hospital in Kitakata, Fukushima on September 30th, a five-hour journey from Tokyo (Figure 1). The patient was accompanied by a nurse but despite challenges during the transfer, including discomfort in a cramped position, the patient exhibited some ability to use a wheelchair and stand up. However, over time, the patient's health further deteriorated, resulting in the accumulation of pleural effusion. After thoracentesis, a slight improvement in dyspnea was noted. Until the day before his passing on November 17th, he could engage in conversations with visiting family members, consistently expressing his strong desire to return to his hometown of Naraha. However, on 16th November, the patient fell into somnolence and passed away before dawn on November 17th.

## 3 Discussion

In this case, a 90-year-old man with COPD, who had been receiving home oxygen therapy for 20 years, was observed to be debilitated by the stress of repeated evacuations after a radiation disaster and died ~8 months after the commencement of evacuation due to respiratory distress. While there was a lack of complete patient information and comprehensive details on the evacuation process because of limited medical records, it was in fact noted that the patient had been affected by repeated evacuation. Thus, he had to be subsequently hospitalized, with an eventual worsening of his condition. Although it cannot be conclusively determined from this case alone, it raises the possibility that severe respiratory distress requiring home oxygen therapy may present a significant risk factor for disaster-related deaths. For patients utilizing home oxygen therapy, the physical and mental burdens associated with disaster evacuation may potentially exceed those faced by the general population, with enduring effects even beyond the immediate evacuation period.

This case indicates the possibility for patients diagnosed with COPD and undergoing home oxygen therapy to experience physical and mental challenges during disasters. Although the patient required 24-h oxygen administration, the sudden mandatory evacuation order placed a great psychological burden on him as he had to move from one evacuation site to another fearing running out of oxygen backup at any time. Moreover, at the time, the fact that the extent of radiation exposure was entirely unknown, and repeated emphasis on the dangers of radiation, coupled with the uncertainty of returning to a familiar place (i.e., the patient's hometown) for an extended period, added to the psychological burden. References from the past literature also mention psychological distress regarding mental health and low-dose radiation exposure after nuclear accidents (30–36). Furthermore, the patient, who normally did not travel far, was repeatedly evacuated for long periods while sitting in an upright position. This and the situation of not being able to get enough rest in an unfamiliar place exacerbated the tremendous physical burden. Prior studies have explored the physical burden of evacuation and the burden of sitting upright in COPD patients (37, 38). This case may further support the notion that forced and prolonged evacuations, particularly to unsuitable locations for patients with disabilities (such as those receiving home oxygen therapy), impose both physical and psychological burdens (14, 15, 39–41). Additionally, the factor of respiratory diseases may also be a contributing factor to an increased risk of mortality, as observed in previous research.

Massive nuclear disasters, characterized by the need for repeated evacuations, may also result in a longer period between the occurrence of the disaster and indirect disaster-related deaths due to the prolonged effects of evacuation compared to a normal crisis (42). In Japan, before the Fukushima Daiichi Nuclear Power Plant accident, the Nagaoka criteria (developed by the city of Nagaoka after the 2004 Niigata Chuetsu-oki Earthquake) were used as standards for certifying disaster-related deaths. According to these criteria, if the period between the occurrence of a disaster and a death exceeds six months, the death is regarded as “less likely to be related to the disaster.” Although evacuations due to the frequent earthquakes often last for only a week to a few months, radiation disasters are characterized by the need for long-term evacuation. In this case, the patient also experienced three to four transfers during the various evacuations within a short span of 6 months. The first evacuation took place immediately after the disaster, while subsequent evacuations took place 6 months later. The prolonged nature of the overall evacuation is a significant distinction between nuclear disasters and other types of calamities. Even a single evacuation imposes physical and psychological burdens on vulnerable individuals. However, the need for repeated evacuations could compound these challenges (39).

Previous studies have suggested the vulnerability of patients to home oxygen therapy during disasters; however, there may be insufficient practical measures currently in place (7, 8, 29). Past research indicates that patients and their families may be unaware of the vulnerability of patients with home oxygen therapy during disasters, thus highlighting the importance of patient education as a future challenge to tackle (29). In the present case, there was no government intervention; instead, patients with home oxygen therapy or their families communicated directly with the manufacturer of the oxygen cylinders, to ensure a continuous oxygen supply. However, difficulties in communication and coordination with the company resulted in patients experiencing daily anxiety. Given such cases, potential areas for improvement could involve the active provision of information and support from governments and healthcare-related institutions. Establishing mechanisms through which the government, equipment manufacturers, and patients can collaborate would help to ensure the wellbeing of patients with home oxygen therapy during disasters. For instance, it is important to jointly develop disaster preparedness guidelines, like deciding in advance where to evacuate based on different scenarios. Additionally, developing a system where real-time location information of patients is shared with governmental agencies and oxygen cylinder companies, or placing portable cylinders in designated evacuation locations in advance, are crucial steps that should be taken.

In Japan, due to privacy concerns, there is no unified database established by relatively large institutions such as national or governmental bodies regarding disaster-related deaths, and the data is not analyzed and disclosed to the public. In this case, case reports are being made with the cooperation of lawyers and bereaved families; however, the data on patients is limited, making it difficult to obtain records based on actual records completely. Additionally, there is no information available on other patients who underwent home oxygen therapy and died in disaster-related incidents, making comparisons impossible. In the future, as the population ages, it is pivotal to establish a comprehensive database on disaster-related deaths and implement disaster measures based on past lessons learned.

## 4 Limitation

Due to incomplete patient information and a lack of comprehensive details on the evacuation process, no definitive conclusions can be drawn from this case alone. This is due to two issues—the absence of a database on disaster-related deaths from a human information protection perspective. Therefore, there is no information on people who died from disaster-related causes and who received home oxygen, making comparisons difficult. Furthermore, the retention period of medical records made it difficult to obtain detailed information on deceased patients. In the future, it is hoped that a disaster-related death database will be established, enabling detailed research on the difficulties faced by patients receiving home oxygen therapy during evacuations in times of disaster, based on information from multiple patients.

## 5 Conclusion

This case reveals the potential for both physical and psychological burdens experienced by vulnerable groups like patients undergoing home oxygen therapy during evacuations in times of disaster. Although it is only a case report and the information is limited, for patients with home oxygen therapy, more severe health impacts are implied, potentially accelerating the onset of adverse outcomes, including premature deaths, especially in cases where evacuations are prolonged, such as in nuclear disasters. Due to the challenge of obtaining prompt public support immediately after a disaster, home oxygen therapy patients may need to prioritize self-help and mutual assistance in their disaster preparedness efforts.

## Data availability statement

The original contributions presented in the study are included in the article/supplementary material, further inquiries can be directed to the corresponding author.

## Ethics statement

The requirement of ethical approval was waived by the Jyoban Hospital of Tokiwa Foundation's Institutional Ethics Committee for the studies involving humans. The studies were conducted in accordance with the local legislation and institutional requirements. The participants provided their written informed consent to participate in this study. Written informed consent was obtained from the individual(s) for the publication of any potentially identifiable images or data included in this article.

## Author contributions

MY: Writing – original draft, Writing – review & editing, Conceptualization. TS: Conceptualization, Writing – review & editing, Writing – original draft. AO: Writing – review & editing. TZ: Writing – review & editing, Funding acquisition. CY: Writing – review & editing, Funding acquisition. SM: Writing – review & editing. MK: Writing – review & editing. YU: Writing – review & editing. HY: Writing – review & editing. KK: Writing – review & editing. HM: Writing – review & editing. MT: Writing – review & editing, Conceptualization, Funding acquisition.
